# Quasi-MOFs in water treatment: Synthesis, characterization, and applications

**DOI:** 10.1016/j.ese.2025.100650

**Published:** 2025-12-20

**Authors:** Xin-Jie Li, Fei Wang, Yuliang Dong, Shih-Hsin Ho, Chong-Chen Wang

**Affiliations:** a Institute of Advanced Materials, Beijing Key Laboratory of Functional Materials for Building Structure and Environment Remediation, Key Laboratory of Urban Stormwater System and Water Environment (Ministry of Education), Beijing University of Civil Engineering and Architecture, Beijing, 100044, China; b Shandong Provincial Key Laboratory of Water and Soil Conservation and Environmental Protection, College of Resources and Environment, Linyi University, Linyi, 276000, China; c State Key Laboratory of Urban-rural Water Resource and Environment, School of Environment, Harbin Institute of Technology, Harbin, 150090, China

**Keywords:** Quasi-MOFs, Defect engineering, Water purification, Catalysis, Adsorption

## Abstract

Metal-organic frameworks (MOFs) are widely investigated for water purification, yet conventional materials are often limited by saturated metal nodes that restrict active-site accessibility and by microporous channels that impede mass transport. Defect engineering provides a means to generate unsaturated metal sites and hierarchical porosity while preserving framework integrity. Quasi-MOFs occupy a distinct position within this landscape, retaining partial long-range order and local coordination environments of the parent MOF while incorporating controlled defects that yield high densities of coordinatively unsaturated sites and multimodal pore structures. In this review, we summarize synthetic strategies that enable precise control of defect type, density, and distribution in quasi-MOFs, including thermal activation, post-synthetic ligand exchange, and modulated coordination approaches. We examine advanced characterization techniques that reveal correlations between engineered defects and enhanced pollutant diffusion and catalytic activation. Applications in adsorptive removal and advanced oxidation/reduction processes are analyzed, highlighting performance advantages derived from improved site accessibility and transport kinetics relative to pristine MOFs. Finally, we discuss persisting challenges, including hydrolytic stability, scalable synthesis, and detailed structure-activity relationships, and outline future directions for translating quasi-MOFs into practical water-treatment technologies.

## Highlights

• Quasi-MOFs retain partial crystallinity with engineered defects and hierarchical porosity.
• Defect strategies expose unsaturated metal sites, enhancing pollutant accessibility.
• Thermal, post-synthetic, and modulated methods enable precise defect control.
• Quasi-MOFs outperform pristine MOFs in adsorption and catalytic water purification.
• Platform advances scalable, stable technologies for aqueous contaminant removal.

## Graphical abstract

**Figure:**
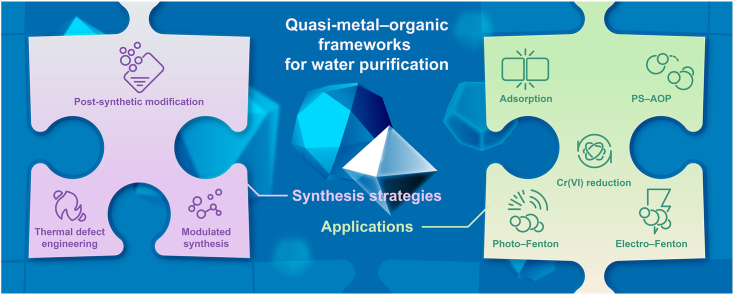

## 1 Introduction

As crystalline nanoporous materials constructed via coordination-driven self-assembly between metal/clusters and multidentate linkers, metal–organic frameworks (MOFs) have garnered significant attention as advanced water purification materials [[1], [2], [3], [4], [5]]. Their inherent modularity enables precise structural customization, allowing for the rational design of metal nodes, ligands, and pore architectures to optimize functionality [6,7]. Nevertheless, two intrinsic limitations hinder their practical applications, especially in the water purification field: (i) highly coordinated frameworks often lead to metal site saturation, limiting their accessibility to interaction with oxidants and target pollutants, thus further inhibiting the generation of reactive oxygen species (ROSs) [8], and (ii) low pore utilization efficiency due to narrow channels or limited porosity hinders the mass transport of pollutants and oxidants [[9], [10], [11], [12]], causing an accumulation of intermediate products that partially block the channels and competitively occupy active sites [[13], [14], [15]]. These two limitations necessitate innovative strategies to activate dormant active sites in existing frameworks [16,17]. Recent advances in targeted defect engineering, in which the controlled introduction of structural imperfections creates accessible active centers while maintaining framework stability, have demonstrated particular promise [[18], [19], [20], [21]].

Emerging as a versatile design paradigm, defect engineering enables the precise modulation of MOF properties through customizable modifications to adsorption capacity [[22], [23], [24]], band structures [[25], [26], [27]], conductivity [28,29], mechanical responses [30,31], and catalytic properties [32,33]. Hierarchical porous MOFs represent a distinct class of defect-engineered materials that strategically incorporate micro-, meso-, and macropores through controlled defect creation (e.g., ligand deficiencies and modulator-induced defects) [[34], [35], [36], [37], [38], [39], [40]]. These materials maintain remarkable structural integrity, exhibiting well-defined powder X-ray diffraction (PXRD) patterns despite containing intentional vacancies, until defect concentration reaches a critical threshold that triggers a complete loss of long-range order and transformation into amorphous MOFs. This transition contrasts with MOF glasses formed by melt-quenching [[41], [42], [43]], which exhibit exclusively short-range coordination. First proposed by Xu's group in 2018, quasi-MOFs emerged as a typical defect MOF [44]. Quasi-MOFs occupy a structural niche in materials chemistry, serving as a transitional phase that bridges the ordered crystalline framework of parent MOFs and their fully transformed derivatives. The as-prepared quasi-MOFs maintain partial crystallinity with locally preserved coordination networks, incorporating strategically engineered defects that create high-density active sites, including unsaturated metal centers and ligand vacancies, as well as hierarchical porosity. This hybrid architecture combines the structural integrity of conventional MOFs with the enhanced functionality typically associated with more disordered materials, offering an optimal balance between framework stability and catalytic and adsorptive performance. The controlled introduction of defects through precise engineering transforms quasi-MOFs into versatile materials that retain a sufficient long-range order for structural robustness [45,46].

The hybrid architecture of quasi-MOFs is notable for combining preserved crystallinity with engineered hierarchical porosity, achieving an optimal balance between structural stability and mass transport efficiency [47,48]. By combining the inherent advantages of traditional MOFs with the enhanced accessibility and diffusion benefits characteristic of defect-engineered materials, quasi-MOFs apply targeted structural modifications, such as partial deligandation to expose open metal centers, the formation of disordered hierarchical pores, and the deliberately introduce functional groups, to achieve accelerated guest transport. Doing so enhances active-site exposure and superior water purification performance [49,50]. These modifications have enhanced the adsorption capacities and reaction rates, thereby overcoming the limitations of traditional MOFs in removing aquatic pollutants.

Despite its remarkable potential, the emerging field of quasi-MOF research remains nascent and underdeveloped [[51], [52], [53], [54], [55], [56]], necessitating a comprehensive review to consolidate it systematically. This critical review summarizes and discusses the following significant issues:

(i) establishing clear conceptual frameworks by delineating quasi-MOFs from related materials;
(ii) synthesizing recent breakthroughs in water treatment applications, including mechanistic insights into pollutant removal and performance comparisons across systems;
(iii) formulating design principles addressing current limitations for next-generation development.

Through a rigorous examination of structure-property-application relationships and the identification of key scientific challenges, this review provides a strategic roadmap for advancing quasi-MOFs as precision-engineered solutions to complex water purification needs.

## 2 Synthesis strategies and characterization techniques of quasi-MOFs

### 2.1 Synthesis strategies of quasi-MOFs

#### 2.1.1 Thermal defect engineering

Thermal defect engineering is the most common strategy for preparing quasi-MOFs [57]. Pyrolysis temperature is a crucial factor influencing structural evolution and preservation [58,59]. At low temperatures, the MOF framework undergoes partial deligandation and node restructuring, generating vacancies and coordinating unsaturated metal sites (CUSs) while retaining part of the original ordered framework. This results in intermediate state characteristics of quasi-MOFs with localized disorder. If the temperature is too high, the framework's long-range order is almost lost, and the material primarily transforms into derivative phases unrelated to the parent topology. Controlled pyrolysis regulates defect density and crystalline retention, enabling the generated CUSs and vacancies to offer highly active adsorption or catalytic sites [[60], [61], [62], [63], [64]], which provide anchoring sites for nanoparticles (NPs) or functional groups. Thus, they significantly enhance the selective removal performance of specific pollutants [65,66].

Equally critical is the pyrolysis atmosphere, in which some inert gases (e.g., N_2_ and Ar) are commonly used instead of air for the synthesis of quasi-MOFs. When pyrolysis is achieved in air, the temperature must be strictly controlled to avoid the complete oxidation of the framework into a single oxide phase [67]. In an oxygen-free environment, pyrolysis helps introduce oxygen vacancies (OVs) into metal-oxygen clusters and inhibits the formation of large oxide phases [68]. By rationally regulating temperature and atmosphere, a controlled balance between framework order and defect density can be achieved, resulting in quasi-MOFs with tunable performance.

The first quasi-MOF material was synthesized with pyrolysis by Xu's group [69]. They heated Au-loaded MIL-101(Cr) at 300 °C in an inert atmosphere to produce Au/Q-MIL-101(Cr). Low-temperature treatment preserved the porosity of MIL-101 while exposing more Cr-O sites than pristine MIL-101, demonstrating enhanced interactions between immobilized Au NPs and inorganic nodes. Thus, low-temperature treatment significantly enhanced CO catalytic oxidation activity, which cannot be achieved with traditional MOFs or oxide materials. Pyrolysis generates high concentrations of vacancy-type defects through thermal decomposition, but limits spatial control over defect locations. It faces challenges in maintaining framework stability and achieving reproducibility.

#### 2.1.2 Post-synthesis modification

Solvent-assisted ligand exchange strategy is a post-synthesis modification method. In this process, pre-synthesized MOFs, acting as elf-sacrificial templates, were immersed in a solvent containing the target ligands to partially replace the original linkers. This *in situ* exchange introduces specific structural defects (i.e., missing or replaced linkers) and exposes additional metal sites or functional groups, while retaining part of the original crystal framework.

Based on solvent-assisted ligand exchange strategy, Luo et al. developed a Zn-based quasi-MOF for lithium-sulfur batteries [70]. The exchanged quasi-MOFs exhibit highly porous, hierarchical structures rich in exposed Zn active sites and uniformly dispersed sulfur. These characteristics result in excellent lithium polysulfide (LiPs) adsorption capacity and enhanced battery performance (high discharge capacity and low capacity-fading rate).

A solvent-assisted ligand exchange strategy introduces chemical substitution defects through solution-mediated, diffusion-controlled mechanisms. This method allows for moderate control over defect concentration and distribution, but may result in incomplete exchange, unwanted side reactions, and the maintenance of structural integrity (e.g., framework decomposition or ligand hydrolysis).

#### 2.1.3 Modulated synthesis

Zhang et al. [71] proposed an “atomized ligand” strategy, which can be categorized as a modulated synthesis approach for generating quasi-MOFs by dynamically controlling ligand–metal coordination. In this method, atomized organic ligands were introduced to metal precursors, resulting in temporally dispersed and incomplete coordination on the surface. Rather than forming fully ordered frameworks, this approach slowed the crystal ripening process and promoted the formation of quasi-MOF clusters rich in CUSs, causing local structural disorder. The resulting materials retained part of the ordered structure of the parent framework while providing a high density of accessible active sites.

When applied to the HKUST-1 system, 0.2 g of H_3_BTC solution (30 mL H_2_O/EtOH) is sprayed onto the Cu(OH)_2_ nanowire film at a rate of ∼0.25 mL s^−1^. After just 60 s of atomization at room temperature, a quasi-HKUST-1 rich in coordinatively unsaturated Cu paddle wheel (CU@CPW) clusters was generated. Compared with fully crystallized HKUST-1, quasi-HKUST-1 exhibits significantly enhanced CO_2_ reduction performance because the CU@CPWs act as catalytically active sites, enabling a more efficient proton-coupled multi-electron transfer reaction than the coordinatively saturated CPW.

Atomized ligand strategy introduces rich CUSs and unsaturated coordinated metal-ligand structural units through rapid partial coordination. This method excels in spatial defect localization, as ligand atomization confines coordination to surface regions. However, challenges remain in precisely controlling atomization kinetics, ensuring uniform ligand distribution, and achieving scalability.

In addition to the previously mentioned methods (Table 1), other strategies can be employed to prepare quasi-MOFs. For example, chemical etching can selectively remove ligands or metal nodes. Plasma treatment and mechanochemistry (e.g., ball milling) can induce local reconstruction and defect accumulation without completely destroying the framework. Template-assisted etching can impart partially selective, functionalized structures to quasi-MOFs. Future research should explore new methods for precisely controlling defect distribution, enhancing uniformity and reproducibility, and facilitating the scalable design and construction of quasi-MOFs.

**Table 1:** Preparation strategy of quasi-MOFs.

Method	Classification	Mechanism	Advantages	Limitations
Controlled pyrolysis	Thermal defect engineering	Partially removing ligands and rearranging nodes under moderate temperature and atmosphere to generate vacancies, coordinating unsaturated metal sites (CUSs), and new-phase nanodomains while partially retaining the framework	Simple and easily tunable method with high reproducibility	Difficult to control the distribution of new phases and requires precise control of temperature and atmosphere
Solvent-assisted ligand exchange	Post-synthesis modification	Partially replacing original ligands with target ligands in a solution to introduce vacancies and new functional groups while retaining part of the framework	Precise tuning of chemical composition and functionality	Diffusion limitations, uneven spatial exchange, and high defect levels, possibly causing instability, limited reproducibility, and scalability
Atomized ligand strategy	Modulated synthesis	Atomizing the ligand solution to dynamically control the spatiotemporal distribution of ligand–metal coordination, suppress complete crystal growth, and form local disorder with high-density CUSs	Efficient and controllable generation of CUSs and a high density of active sites	Highly sensitive to conditions such as atomized droplet size and flow rate, making it challenging to scale up and difficult to control uniformity

### 2.2 Characterization techniques of quasi-MOFs

**Fig. 1:**
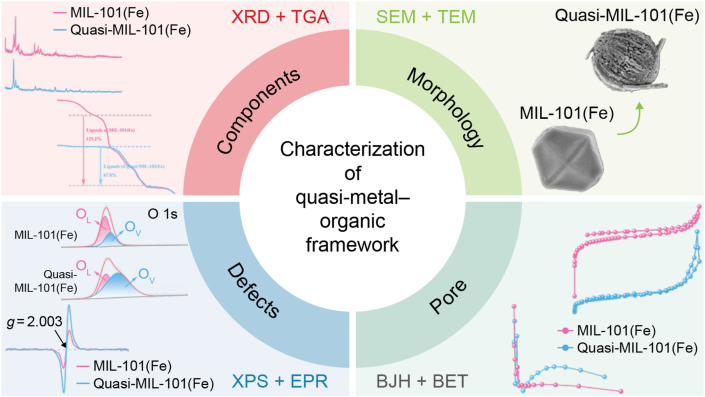
Basic characterization methods of quasi-metal–organic frameworks. XRD, X-ray diffraction; TGA, thermogravimetric analysis; SEM, scanning electron microscopy; TEM, transmission electron microscopy; XPS, X-ray photoelectron spectroscopy; EPR, electron paramagnetic resonance; BJH, Barrett–Joyner–Halenda method; BET, Brunauer–Emmett–Teller theory.

The unique structures of quasi-MOFs require comprehensive and multi-scale characterisation to detect their crystallinity, defect distribution, surface chemical properties, porosity, and the local coordination environment of metal centers (Fig. 1).

Powder X-ray diffraction analysis is widely used to monitor structural changes during pyrolysis [13]. In quasi-MOFs, the intensity decrease and broadening of the characteristic peaks of the pristine MOF indicate a reduction in crystallinity and orderliness, implying partial deligandation from the pristine MOF structure. Shen et al. [72] systematically investigated the thermal evolution of HKUST-1 using PXRD, observing critical structural transitions during pyrolysis. Their analysis demonstrated preserved crystallinity at 300 °C, with characteristic peak intensities progressively diminishing upon heating until only Cu-related peaks remained at 400 °C, indicating a complete framework collapse. These findings enabled precise temperature control (300 °C) for synthesizing Cu-nanowire@Quasi-MOF while maintaining partial crystallinity, with PXRD providing crucial insights into the controlled transformation from pristine MOF to defect-engineered quasi-MOF structure. Comprising coexisting crystalline and amorphous domains, the structural duality of quasi-MOFs presents unique characterization challenges. While XRD patterns typically show peak broadening or attenuation, these features should not be automatically interpreted as complete framework degradation. This complexity underscores the need for multimodal characterization approaches combining diffraction, spectroscopy, and microscopy to accurately distinguish between desirable defect formation and detrimental structural collapse in quasi-MOF systems.

Thermogravimetric analysis (TGA) contributes to determining the appropriate pyrolysis temperature and estimating ligand loss [73]. Li et al. [74] analyzed the pyrolysis process of MIL-101(Fe) via TGA, revealing three weight-loss stages corresponding to solvent loss, ligand degradation, and framework collapse. The coordination number of Fe-O clusters was derived from mass loss during the ligand removal process, decreasing from 5.4 in pristine MIL-101(Fe) to 2.9 in Q350-MIL-101(Fe). A quantitative analysis of ligand loss was achieved. While TGA provides crucial quantitative data on mass changes during thermal treatment, this technique exhibits several inherent limitations for characterizing quasi-MOF formation. For example, the measured weight loss profiles represent bulk macroscopic behavior and cannot provide the following: (i) spatial resolution to identify localized structural changes, (ii) chemical specificity to differentiate between ligand decomposition and metal cluster alterations, and (iii) mechanistic insights into whether observed mass losses correspond to linker removal, cluster decomposition, or framework reorganization. These constraints necessitate complementary characterization approaches, such as *in situ* spectroscopy or mass spectrometry, to fully elucidate the complex structural transformations occurring during quasi-MOF synthesis.

Fourier transform infrared (FTIR) and Raman spectroscopy spectra can detect changes in metal–ligand bonding. The broadening or weakening of characteristic vibrational peaks can further indicate partial deligandation and disruption of the coordination structure, in which shifts in the vibrational frequencies of the metal–oxygen clusters or the carboxylate linkers (e.g., asymmetric and symmetric COO^−^ stretches) serve as direct probes of the coordination environment. Ai et al. [75] observed a broadening of the carboxyl group peak near 1578 cm^−1^ in the FTIR spectrum of Ni_2_P@quasi-Ni-BDC, which was consistent with the PXRD results. This broadening indicated partial deligandation and confirmed the formation of a quasi-MOF. Cui et al. [76] observed a characteristic Raman band at 3598 cm^−1^ in Co/MnO_*x*_@quasi-MOF-74 attributed to Co^2+^–OH coordination, which confirmed an altered local environment of metal sites and a quasi-MOF structure. During pyrolysis, organic linkers are typically converted into a carbonaceous matrix, and Raman spectroscopy is highly sensitive to the structural features of carbon-based materials. In future research, Raman spectroscopy could characterize the extent of disorder and the size of the remaining sp^2^-hybridized carbon [77,78]. However, vibrational spectra are often affected by overlapping signals and lack spatial resolution. Minor changes in the coordination structure may be masked by complex background signals, limiting their ability to identify quasi-MOFs.

N_2_ adsorption–desorption isotherms are used to evaluate pore size evolution. Quasi-MOFs often exhibit increased pore size due to framework expansion or partial collapse, but the trend in specific surface areas varies situationally. In some cases, the increased surface area may be attributed to pore expansion and improved connectivity, while in others, pore blockage occurs due to aggregation or framework damage. Bagheri et al. [79] observed a decrease in the Brunauer–Emmett–Teller (BET) surface area of quasi-HKUST-1 (QH-240) with increasing pyrolysis temperature. Micro- and mesopores forming in the structure were attributed to defects or vacancies resulting from partial ligand removal. Notably, the presence of mesopores in quasi-MOFs may yield different BET interpretations compared to those of pristine MOFs, which typically contain only micropores. In accordance with International Union of Pure and Applied Chemistry guidelines, the BET method should be applied cautiously to materials displaying Type I adsorption isotherms, a characteristic feature of microporous solids. The calculated surface area should be considered apparent rather than as a true, physically accessible value. Nevertheless, this calculation is a useful parameter for material comparison and quality control [80]. While BET analysis provides valuable quantitative data on the overall porosity and specific surface area of quasi-MOFs, this bulk characterization technique has inherent limitations in mechanistic interpretation. The measured parameters represent ensemble averages across the entire sample, making it impossible to differentiate various structural alterations. These alterations include framework collapse, partial ligand removal, and particle aggregation phenomena, all of which could contribute to the observed changes. This fundamental constraint underscores the necessity of complementing BET measurements with other characterization methods, such as electron microscopy or X-ray scattering, to gain a more comprehensive understanding of the specific structural modifications that occur during quasi-MOF formation.

X-ray photoelectron spectroscopy (XPS) is applied to analyze the surface elemental composition and metal valence state. In quasi-MOFs, reduced C/N-to-metal ratio and changed metal oxidation state reflect partial ligand loss and the formation of CUSs. Xia et al. [81] observed a partial reduction of Fe^3+^ to Fe^2+^ during the pyrolysis of NH_2_-MIL-101(Fe), accompanied by a relative decrease in the intensity of alkyl and carboxyl peaks and an increase in Fe content, confirming the generation of CUSs. While XPS provides valuable surface chemical information about quasi-MOFs, its inherent surface sensitivity (typically probing <10 nm depth) presents significant limitations for comprehensive material characterization. The technique's shallow sampling depth results in the following: (i) surface oxidation or contamination may dominate spectra, (ii) bulk compositional changes remain undetected, and (iii) depth-dependent structural gradients are obscured. Consequently, XPS data should be carefully corroborated with bulk-sensitive techniques, such as XRD and bulk elemental analysis, to obtain a full understanding of the material's structural evolution during defect engineering processes.

Electron paramagnetic resonance (EPR) is widely used to identify OVs in quasi-MOFs. The defect-rich coordination environment caused by partial deligandation often generates paramagnetic centers, resulting in characteristic EPR signals [82]. Li et al. [74] established that quasi-MIL-101(Fe) exhibited a strong EPR signal corresponding to a g-factor of 2.003, which was consistent with the enhanced O 1s signal observed in XPS results and further confirmed the formation of OVs. While EPR is highly sensitive for detecting paramagnetic centers in quasi-MOFs, it faces significant challenges in quantitative defect analysis. The primary limitations include the following: (i) an inability to directly correlate signal intensity with absolute defect concentrations due to unknown transition probabilities, (ii) difficulty distinguishing different types of paramagnetic defects with similar *g*-factors, and (iii) a lack of spatial resolution to map defect distributions in the material. These constraints necessitate the use of complementary quantitative techniques, such as elemental analysis or titration methods, combined with EPR, to establish reliable structure–property relationships in defect-engineered quasi-MOFs.

Mössbauer spectroscopy enabled a precise determination of the oxidation state and local environment of iron, making it valuable for Fe-containing quasi-MOFs. In addition, extended X-ray absorption fine structure (EXAFS) spectroscopy can reveal variations in bond lengths and coordination asymmetry. Chang et al. [83] determined that EXAFS and the Mössbauer spectra of quasi-MIL-100(Fe) exhibited increased electron density at Fe sites. This increased electron density facilitated the generation of various ROSs, contributing to Q350-MIL-100(Fe)'s superior oxidation ability and reaction rate. However, these characterization techniques present notable constraints: Mössbauer spectroscopy is inherently element-specific and applicable only to select isotopes, such as ^57^Fe, while EXAFS measurements demand synchrotron radiation sources and sophisticated modeling approaches that can mask the structural signatures of disordered components in quasi-MOFs.

Electron microscopy techniques, including scanning electron microscopy (SEM) and high-resolution transmission electron microscopy (HRTEM), aid in visualizing morphology, particle size, and local structural order [84,85]. SEM reveals particle size distribution, aggregation behavior, and surface roughness after pyrolysis [86]. HRTEM enables the direct observation of lattice fringe disruptions and metal cluster formation, providing nanoscale evidence of framework distortion and partial crystallinity preservation in quasi-MOFs [87]. It is necessary to explicitly note its frequent combination with energy dispersive X-ray spectroscopy (EDS) or electron energy loss spectroscopy (EELS). These complementary techniques enable nanoscale elemental mapping, which is essential for confirming the spatial distribution of metal nodes, identifying dopant locations, and verifying the formation of new phases in quasi-MOF structures. Tsumori et al. [44] found uniformly dispersed Au nanoparticles (<3 nm) in MIL-101 by High-Angle Annular Dark-Field Scanning Transmission Electron Microscopy, while EDS mapping confirmed the homogeneous distribution of Au with Cr, C, and O, evidencing the successful formation of Au/quasi-MIL-101 with well-retained dispersion stability.

Despite significant progress, conventional techniques remain limited in terms of spatial and temporal resolution. Emerging techniques, such as confocal fluorescence microscopy, atomic force microscopy, fluorescence lifetime imaging microscopy, positron annihilation lifetime spectroscopy (PALS), and solid-state nuclear magnetic resonance (NMR), offer powerful capabilities to probe local defects, electronic environments, and dynamic processes in quasi-MOFs. These approaches are expected to unveil multiscale structure–property relationships and guide future rational design [[88], [89], [90]].

## 3 Quasi-MOFs for water purification

### 3.1 Defect-engineered quasi-MOFs for enhanced adsorptive removal of aqueous pollutants

While Wang's group and have demonstrated the potential of pristine MOFs as water purification adsorbents [[91], [92], [93]], the limited availability of accessible binding sites has often hindered their effectiveness. Although these materials exhibit excellent aqueous stability, organic linkers or solvent molecules typically occupy the metal coordination sites that are crucial for contaminant binding [[94], [95], [96], [97], [98]]. This limitations particularly evident in phosphate removal, where blocked active sites in conventional MOFs hinder strong metal–phosphate coordination [99]. Quasi-MOFs overcome these limitations through deliberate defect engineering, which creates two main advantages: the exposure of previously inaccessible metal coordination sites and the generation of new defect-associated functional groups. These structural modifications significantly enhance adsorption performance by providing additional binding sites while maintaining the framework's stability, offering a promising means of efficient water treatment.

When using quasi-MOFs for the adsorption of organic contaminants, pore size, defect density, and chemical functionality were significant in determining adsorption performance [[100], [101], [102]]. Excessively small pore sizes could hinder adsorption capacity by restricting molecular access [103]. Partial deligandation introduced hierarchical micro- and mesoporous structures, facilitating mass transfer and allowing large contaminant molecules to access the aperture. Generating OVs and CUSs activated previously inaccessible high-energy binding sites, greatly increasing adsorption capacity [104,105]. Moreover, the abundance of reactive and selective binding sites in quasi-MOFs can improve the selective adsorption of target pollutants.

Wang et al. accomplished the controlled pyrolysis of NH_2_-MIL-125(Ti) at 250 °C under a nitrogen atmosphere [48], which strategically generated surface OVs while preserving framework integrity. The resulting defect-engineered material exhibited a hierarchical pore structure combining micropores and mesopores with two significant modifications: the creation of unsaturated Ti^4+^ centers and the introduction of oxygen defect sites. These structural alterations synergistically enhanced the material's adsorption performance through multiple mechanisms, such as strengthened electrostatic interactions, additional coordination sites, and improved diffusion pathways. The quasi-MOF NH_2_-MIL-125@250 °C demonstrated remarkable adsorption capacities of 262 mg g^−1^ for indole and 251 mg g^−1^ for quinoline, setting a new benchmark for nitrogen-containing compound removal. This seminal work confirmed the concept of defect-enhanced adsorption and revealed the critical role of carefully controlled thermal modification in optimizing MOF adsorbents for water treatment applications.

**Fig. 2:**
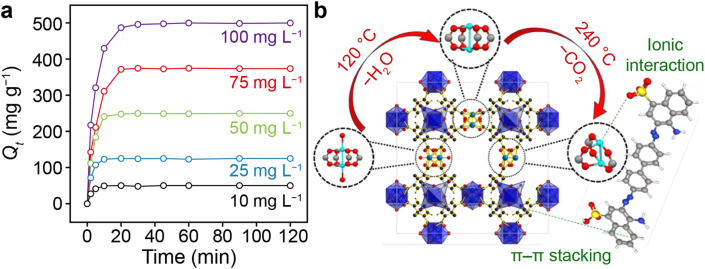
**a**, Influence of congo red (CR) concentrations on the adsorption ability (*Q*_*t*_) over QH-240. **b**, Proposed adsorption mechanism of CR over QH-240. Adapted from Ref. [47]. Copyright 2023. Elsevier.

Rouhani et al. [47] developed a quasi-HKUST-1 material (QH-240) containing abundant open Cu sites and a hierarchical pore network through controlled thermal modification. This modification achieved the efficient, rapid removal of various macromolecular dyes, especially Congo red, through electrostatic interactions, pore filling, and π–π stacking (Fig. 2). QH-240 achieved a remarkable capacity of 715 mg g^−1^ toward Congo red in just 15 min, a tenfold improvement over pristine HKUST-1. This improvement can be attributed to its uniquely balanced combination of increased pore dimensions facilitating macromolecular diffusion and preserved high-density active sites, which ensure strong binding. These findings highlight how targeted defect engineering can transform conventional MOFs into high-performance adsorbents for challenging macromolecular pollutants.

Quasi-MOFs demonstrate adsorption performance superior to their pristine counterparts owing to their structural features, including exposed metal sites, OVs, and hierarchical porosity. These characteristics synergistically enhance adsorption capacity and kinetics, particularly for challenging contaminants such as large organic molecules (e.g., dyes and pharmaceuticals) and trace-level pollutants. The increased accessibility of active sites and optimized pore structures facilitates stronger interactions, enabling efficient removal even at low concentrations. However, a deeper mechanistic understanding based on direct experimental evidence (e.g., *in situ* spectroscopy) or computational validation (e.g., density functional theory calculations) is still needed. Furthermore, precise structure–performance correlations remain underexplored, necessitating future research to guide the rational design of quasi-MOFs for targeted pollutant removal. Addressing these gaps will advance their practical application in water purification.

### 3.2 Environmental catalysis with quasi-MOFs: mechanisms and applications

Quasi-MOFs demonstrate catalytic performance superior to that of conventional MOFs [106,107], primarily due to their abundant CUSs and structural vacancies. Notably, OVs significantly enhance light absorption and modulate electronic structures by narrowing band gaps and creating intermediate energy levels to facilitate photon capture and charge separation [[108], [109], [110], [111]]. The high-density active sites on quasi-MOF surfaces effectively concentrate water molecules and pollutants at metal centers, enabling rapid redox transformations [[112], [113], [114]]. Beyond their adsorption capabilities, quasi-MOFs exhibit exceptional catalytic potential due to their hierarchical pore structures and large specific surface areas, which facilitate efficient reactant diffusion and substantially accelerate reaction kinetics. This combination of abundant active sites and optimized multiscale porosity makes quasi-MOFs particularly promising for applications in advanced electrocatalysis and oxidation and reduction processes, outperforming traditional MOFs in efficiency and reaction rates.

#### 3.2.1 Persulfate-based advanced oxidation processes (AOPs) enhanced by defect engineering

**Fig. 3:**
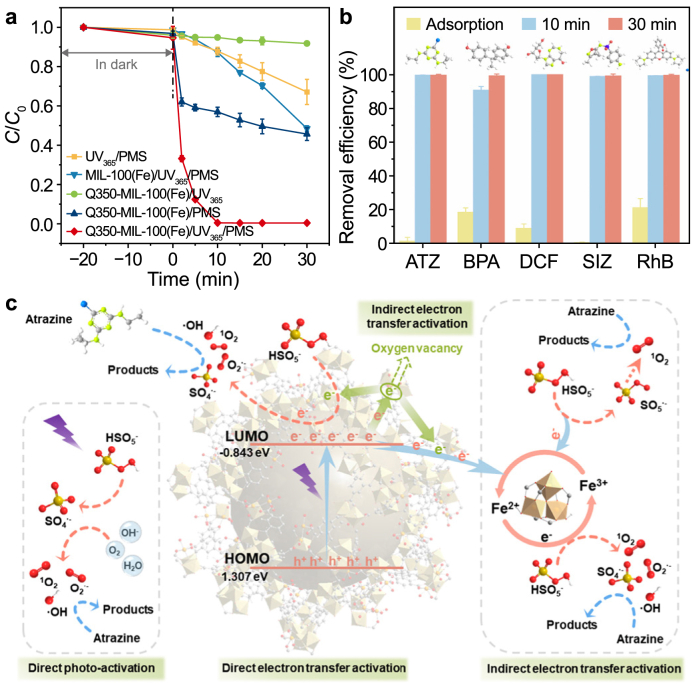
**a**, Degradation efficiency of atrazine (ATZ) in different systems. *C*/*C*_0_ represents the normalized concentration, where *C*_0_ and *C* are the initial concentration and the concentration at time *t*, respectively. **b**, Degradation abilities of Q350-MIL-100(Fe) toward various pollutants. **c**, Mechanism diagram of ATZ degradation by Q350-MIL-100(Fe)/UV_365_/PMS system. PMS, peroxymonosulfate; BPA, bisphenol A; DCF, diclofenac; SIZ, sulfisoxazole; RhB, rhodamine B; LUMO, lowest unoccupied molecular orbital; HOMO, highest occupied molecular orbital. Adapted from Ref. [83]. Copyright 2025. Springer Nature.

Chang et al. synthesized Q350-MIL-100(Fe) by pyrolyzing MIL-100(Fe) for the efficient degradation of atrazine (ATZ) by activating peroxymonosulfate (PMS) (Fig. 3) [83]. The iron source for synthesizing MIL-100(Fe) was innovatively recovered from waste stainless steel pickling wastewater, greatly enhancing the environmental sustainability of the quasi-MOF production process. Beyond the pristine MIL-100(Fe), the enlarged pore size (2.4 nm) facilitates a mass transfer process, allowing ATZ molecules to enter the framework of Q350-MIL-100(Fe) easily. EXAFS and Mössbauer spectroscopy analyses confirmed an increase in electron density and asymmetric coordination at Fe sites, demonstrating an enhanced electron donation capability that significantly boosts PMS activation efficiency. These electronic modifications facilitate rapid ROS generation, improving oxidative capacity and reaction kinetics over those of conventional Fe-MOFs. As prepared, Q350-MIL-100(Fe) exhibited exceptional stability and efficiency, achieving complete degradation (100 %) of ATZ during continuous operation for 96 h in an immobilized reactor system. This study successfully establishes an eco-friendly paradigm for fabricating high-efficiency quasi-MOF catalysts, offering both environmental and technological benefits.

Khojastegi et al. [115] developed a MnO_2_-incorporated quasi-MOF (MnO_2_@Q-MOF) through the nitrogen gas-protected thermal reduction of KMnO_4_ within MIL-53(Fe) at 300 °C, which simultaneously removed phthalate linkers to create accessible pores while preserving the framework's structural integrity, thus generating abundant interfacial sites for synergistic MnO_2_-MOF interactions. Incorporating Mn also facilitated the formation of a channel for hydroxyl ion transfer between Fe and Mn, significantly enhancing the adsorption and activation of PMS to degrade methylene blue (MB) under visible light. Dominated by ^1^O_2_, the degradation process exhibits a high affinity for electron-rich organic compounds, such as MB, which contain unsaturated bonds and heteroatoms, enabling efficient degradation. This research offers a valuable reference for exploring the potential of synergistic redox interactions between bimetallic centers in quasi-MOFs to regulate ROSs and promote the selective removal of pollutants.

#### 3.2.2 Photo-Fenton-Like reaction boosted by oxygen vacancies

**Fig. 4:**
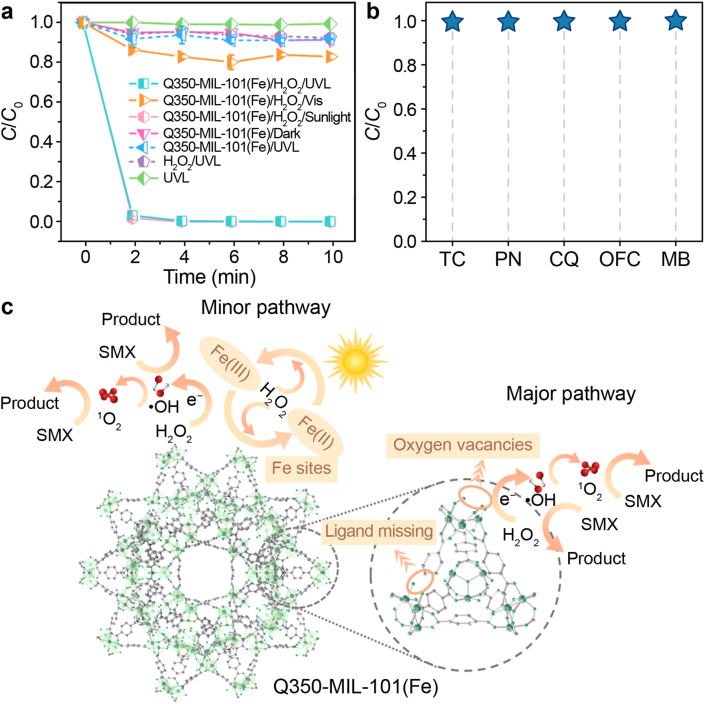
**a**, Degradation efficiencies of sulfamethoxazole (SMX) in different systems. **b**, Degradation performances of Q350-MIL-101(Fe) toward various pollutants. *C*/*C*_0_ represents the normalized concentration, where *C*_0_ and *C* are the initial concentration and the concentration at time *t*, respectively. **c**, Mechanism diagram of SMX degradation by Q350-MIL-101(Fe)/UV/H_2_O_2_ system. UVL, ultraviolet light; TC, tetracycline; PN, phenanthrene; CQ, chloroquine; OFC, ofloxacin; MB, methylene blue. Adapted from Ref. [74]. Copyright 2025. Elsevier.

Li et al. synthesized Q350-MIL-101(Fe) through the controlled pyrolysis of MIL-101(Fe), demonstrating exceptional efficacy in sulfamethoxazole (SMX) removal via a photo-Fenton process (Fig. 4) [74]. The introduction of OVs narrowed the band gap, enhancing light absorption and creating additional active sites, which significantly boosted catalytic performance. Structural characterization revealed an expansion of pore size from 2.2 nm in the parent MIL-101(Fe) to 5.2 nm, facilitating improved mass transport. The catalyst achieved 100 % SMX removal within 84 h of continuous operation, with mechanistic studies identifying •OH and ^1^O_2_ as the predominant reactive species. These findings underscore the material's practical potential while advancing the fundamental understanding of defect engineering in quasi-MOFs for enhanced photo-Fenton applications.

#### 3.2.3 Electro-Fenton catalysis enhanced by defect engineering in quasi-MOFs

**Fig. 5:**
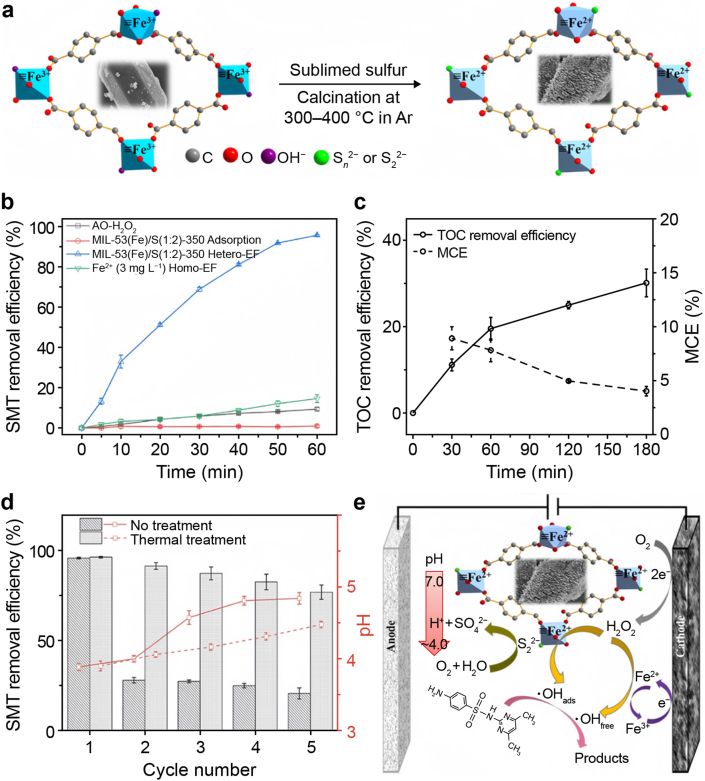
**a**, The evolution of MIL-53 (Fe) structure during pyrolysis. **b**, Sulfamethazine (SMT) degradation efficiency in different Homo-EF processes. **c**, Total organic carbon (TOC) and mineralization current efficiency (MCE) degradation efficiencies of MIL-53(Fe)/S(1:2)350/Hetero EF process. **d**, Sequential testing of MIL-53(Fe)/S(1:2)350 as a function of pH. **e**, Mechanism diagram of MIL-53(Fe)/S(1: 2)350/various EF processes. AO, anodic oxidation; EF, electro-Fenton. Adapted from Ref. [116]. Copyright 2022. Elsevier.

Du et al. [116] developed a sulfur-modified quasi-MIL-53(Fe) catalyst through controlled pyrolysis under an Ar atmosphere (Fig. 5), which exhibited remarkable electro-Fenton performance for sulfamethazine (SMT) degradation. The synthesized material featured a unique 2D nanosheet morphology (∼50 nm thickness) with a significantly enlarged surface area. Sulfur doping achieved a reduction of Fe^3+^ to Fe^2+^ and the introduction of hydroxyl groups in Fe-O chains, substantially increasing Fe^2+^ content. Optimal MIL-53(Fe)/S(1:2)-350 demonstrated exceptional pH adaptability, achieving 95.8 % SMT removal at neutral pH (7.0) with a reaction rate consistently 16-fold higher than conventional homogeneous electro-Fenton systems. Mechanistic investigations through EPR and radical quenching experiments indicated that surface-adsorbed hydroxyl radicals (•OH_ads_) served as the primary reactive species, while free •OH played a secondary role. Notably, the catalyst exhibited outstanding stability and regenerability through simple sulfidation, with its turnover frequency (TOF_d_ = 0.48 L g^−1^ min^−1^) surpassing commercial FeS_2_ by 6.8 times. This work provides fundamental insights into defect-mediated reaction mechanisms and establishes a practical strategy for developing pH-universal quasi-MOF catalysts for advanced wastewater treatment applications.

#### 3.2.4 Defect-mediated photocatalytic reduction of Cr(VI) by quasi-MOFs

**Fig. 6:**
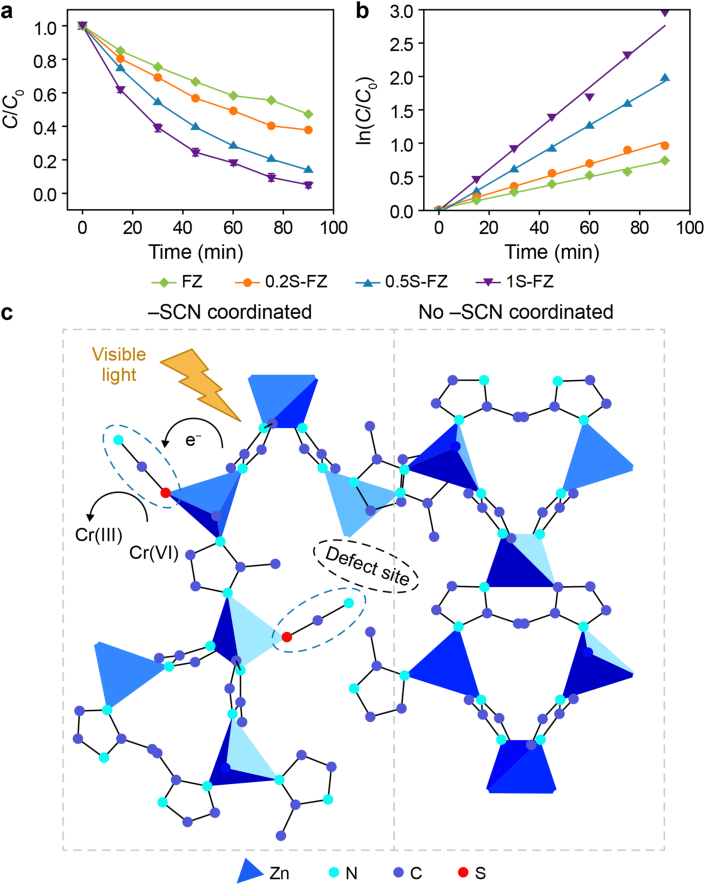
**a**, Photocatalytic reduction of Cr(VI) over different catalysts. **b**, Kinetic analysis based on a pseudo-primary fitting model. *C*/*C*_0_ represents the normalized concentration, where *C*_0_ and *C* are the initial concentration and the concentration at time *t*, respectively. **c**, Photocatalytic mechanistic diagram of 1S-FZ samples. –SCN, thiocyano-. Adapted from Ref. [117]. Copyright 2024. Elsevier.

To overcome the electron utilization limitations in Cr(VI) photocatalytic reduction, Zhang et al. [117] developed an innovative strategy of incorporating thiocyanate (–SCN) groups into an Fe–O cluster-sensitized ZIF-L framework through solvent-assisted ligand exchange (Fig. 6). This modification demonstrated that the introduction of structural defects established stable electron transfer pathways. The strongly nucleophilic sulfur atoms in –SCN groups served as additional catalytic centers, significantly enhancing interfacial charge separation [118,119]. The optimized SCN-ZIF-L photocatalyst demonstrated exceptional performance, achieving a 94.8 % Cr(VI) reduction under visible light irradiation in a neutral KSCN solution without requiring additional sacrificial agents. It also exhibited a 2.8-fold increase in the reaction rate constant compared to its unmodified counterpart. This breakthrough highlights the potential of defect engineering in quasi-MOFs to develop highly efficient, scavenger-free photocatalytic systems for heavy metal remediation, with particular significance for Cr(VI) removal under environmentally relevant neutral pH conditions.

In summary, quasi-MOFs have emerged as highly promising catalysts for advanced oxidation and reduction reactions, offering great potential for the rational design of efficient and sustainable catalytic systems for water purification (Table 2).

**Table 2:** Summary of the performance of different quasi-MOFs in various adsorption and catalytic processes.

Quasi-MOF	MOF precursor	Preparation methods	Pollutant concentration (mg L^−1^)	Reaction type	Reaction conditions	Degradation efficiency/adsorption capacity	Reference
NH_2_-MIL-125@250 °C	NH_2_-MIL-125	Pyrolysis	IND, QUI (1500)	Adsorption	Catalyst (0.67 g L^−1^)	262; 251 mg g^−1^ (6 h)	[48]
QH-240	HKUST-1	Pyrolysis	CR (150)	Adsorption	Catalyst (0.2 g L^−1^)	715 mg g^−1^ (120 min)	[47]
Q-M801-280	MOF-801	Pyrolysis	Phosphate (50)	Adsorption	Catalyst (0.1 g L^−1^)	415 mg g^−1^ (120 min)	[99]
Q-M303-450	MOF-303	Pyrolysis	Phosphate (50)	Adsorption	Catalyst (0.1 g L^−1^)	488 mg g^−1^ (120 min)	[120]
Q-ZIF-67-SH	ZIF-67	Pyrolysis	Hg(II) (1000)	Adsorption	Catalyst (0.05 g L^−1^)	994 mg g^−1^ (60 min)	[121]
MFe⁃250	MIL-53(Fe)	Pyrolysis	MB (20)	PMS–AOP	Catalyst (0.2 g L^−1^); H_2_O_2_ (4.9 mmol L^−1^); visible-light	99 % (90 min)	[122]
Q350-MIL-100(Fe)	MIL-100(Fe)	Pyrolysis	ATZ (5)	PMS–AOP	Catalyst (0.2 g L^−1^); PMS (0.1 mmol L^−1^); UV-light	100 % (10 min)	[83]
MnO_2_@Q-MOF	MIL-53(Fe)	Pyrolysis	MB (50)	PMS–AOP	Catalyst (0.15 g L^−1^); PMS (2 mmol L^−1^); dark	100 % (120 min)	[115]
Q350-MIL-101(Fe)	MIL-101(Fe)	Pyrolysis	SMX (10)	Photo–Fenton	Catalyst (0.2 g L^−1^); H_2_O_2_ (3.92 mmol L^−1^); UV-light	100 % (4 min)	[74]
MIL-53(Fe)/S (1:2)-350	MIL-53(Fe)	Pyrolysis	SMT (10)	Electro–Fenton	Catalyst (0.1 g L^−1^); current, 25.0 mA	95.8 % (60 min)	[116]
1S-FZ	ZIF-L	Modulated synthesis	Cr(VI) (30)	Reduction	Catalyst (0.5 g L^−1^), light source (300 W Xe lamp)	94.8 % (90 min)	[117]

## 4 Conclusion and perspectives

As an emerging class of defective MOFs, quasi-MOFs are still in the early stages of performance exploration. This review provides a comprehensive overview of synthesis strategies, characterization techniques, and recent advances in the application of quasi-MOFs for water purification. Despite significant progress in eliminating catalytic aqueous pollutants from water, several challenges must be addressed in future research.

(1) The current transformation of MOFs into quasi-MOFs remains largely empirical, highlighting a critical need to develop universal synthetic strategies and precursor selection criteria that account for diverse structural parameters. Future research must systematically investigate how primary MOF characteristics, such as metal node identity, ligand stability, and framework topology, govern the dynamics of defect formation and structural evolution, enabling the rational design of quasi-MOFs with tailored properties rather than serendipitous discovery of them. This fundamental understanding will enable researchers to predictably engineer defect types, densities, and distributions, thereby optimizing material performance for specific applications.
(2) The catalytic and adsorption efficiency of quasi-MOFs is fundamentally governed by defect characteristics, such as type, density, and spatial arrangement; however, achieving precise control over these parameters presents a significant challenge in terms of materials. Advancing this field requires developing synthetic methods for tunable, reproducible defect engineering while maintaining framework stability and conducting fundamental studies to elucidate the relationships between defect chemistry and properties in promising materials.
(3) Traditional techniques, such as EPR and XPS, provide limited insight into defect sites and their impact on material properties. Future studies should integrate advanced tools, including PALS, pair distribution function analysis, and *in situ* solid-state NMR with multiscale computational modeling, to more accurately reveal structure–property relationships and mechanism pathways in quasi-MOFs.
(4) Current quasi-MOF synthesis relies heavily on energy-intensive pyrolysis and hazardous solvents, which pose environmental concerns. Future research should prioritize the development of eco-friendly alternatives using renewable solvents, low-energy processes, and sustainable precursors to enable greener production while maintaining optimal material performance.
(5) Moreover, most reported quasi-MOF studies are still limited to laboratory-scale demonstrations under ideal conditions. To bridge the gap between laboratory results and real-world applications, exploring pilot-scale synthesis, long-term aqueous stability testing, and integration into practical water treatment systems is necessary.

Recent advances in quasi-MOF research have revolutionized defect engineering, creating unprecedented opportunities for water purification technologies. These breakthroughs have demonstrated remarkable potential in addressing complex water contamination challenges. By systematically addressing current challenges in synthesis, characterization, and scalability, quasi-MOF materials with enhanced performance, durability, and environmental compatibility will deliver innovative, cost-effective solutions to global water security challenges.

## CRediT authorship contribution statement

**Xin-Jie Li:** Writing – original draft, Conceptualization. **Fei Wang:** Writing – original draft, Resources, Conceptualization. **Yuliang Dong:** Writing – review & editing, Resources. **Shih-Hsin Ho:** Writing – review & editing, Supervision. **Chong-Chen Wang:** Writing – review & editing, Supervision, Conceptualization.

## Declaration of interest competing

The authors declare that they have no known competing financial interests or personal relationships that could have appeared to influence the work reported in this paper.

Dr. Shih-Hsin Ho, the Associate Executive Editor-in-Chief of *Environmental Science and Ecotechnology*, was not involved in the editorial review or the decision to publish this article.
